# Immunosuppressive effect of mesenchymal stem cells on lung and gut CD8^+^ T cells in lipopolysaccharide‐induced acute lung injury in mice

**DOI:** 10.1111/cpr.13028

**Published:** 2021-03-19

**Authors:** Yanping Xu, Jiaqi Zhu, Bing Feng, Feiyan Lin, Jiahang Zhou, Jingqi Liu, Xiaowei Shi, Xuan Lu, Qiaoling Pan, Jiong Yu, Ying Zhang, Lanjuan Li, Hongcui Cao

**Affiliations:** ^1^ State Key Laboratory for the Diagnosis and Treatment of Infectious Diseases Collaborative Innovation Center for Diagnosis and Treatment of Infectious Diseases The First Affiliated Hospital Zhejiang University School of Medicine Hangzhou City China; ^2^ National Clinical Research Center for Infectious Diseases Hangzhou City China; ^3^ Zhejiang Provincial Key Laboratory for Diagnosis and Treatment of Aging and Physic‐chemical Injury Diseases Hangzhou City China

**Keywords:** acute lung injury, gut bacterial communities, gut‐lung axis, immunoregulation, mesenchymal stem cells

## Abstract

**Objectives:**

Acute lung injury (ALI) not only affects pulmonary function but also leads to intestinal dysfunction, which in turn contributes to ALI. Mesenchymal stem cell (MSC) transplantation can be a potential strategy in the treatment of ALI. However, the mechanisms of synergistic regulatory effects by MSCs on the lung and intestine in ALI need more in‐depth study.

**Materials and methods:**

We evaluated the therapeutic effects of MSCs on the murine model of lipopolysaccharide (LPS)‐induced ALI through survival rate, histopathology and bronchoalveolar lavage fluid. Metagenomic sequencing was performed to assess the gut microbiota. The levels of pulmonary and intestinal inflammation and immune response were assessed by analysing cytokine expression and flow cytometry.

**Results:**

Mesenchymal stem cells significantly improved the survival rate of mice with ALI, alleviated histopathological lung damage, improved intestinal barrier integrity, and reduced the levels of inflammatory cytokines in the lung and gut. Furthermore, MSCs inhibited the inflammatory response by decreasing the infiltration of CD8^+^ T cells in both small‐intestinal lymphocytes and Peyer's patches. The gut bacterial community diversity was significantly altered by MSC transplantation. Furthermore, depletion of intestinal bacterial communities with antibiotics resulted in more severe lung and gut damages and mortality, while MSCs significantly alleviated lung injury due to their immunosuppressive effect.

**Conclusions:**

The present research indicates that MSCs attenuate lung and gut injury partly via regulation of the immune response in the lungs and intestines and gut microbiota, providing new insights into the mechanisms underlying the therapeutic effects of MSC treatment for LPS‐induced ALI.

## Graphical abstract

This study focuses on the protective effects of mesenchymal stem cells (MSCs) in the lung and gut of Lipopolysaccharide‐induced acute lung injury mice. The data suggest that MSCs improved intestinal barrier function and reduced intestinal infiltration of CD8^+^ T cells. Further, depletion of intestinal bacterial communities with antibiotics significantly improved the therapeutic effects of MSCs.

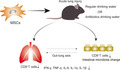

## 1 INTRODUCTION

Sepsis‐related acute lung injury (ALI), the early pathophysiological change in acute respiratory distress syndrome (ARDS), has a high mortality rate due to lack of effective therapies.^1^, ^2^ ALI/ARDS can progress to multiple organ failure, which is characterized by increased lung permeability, pulmonary oedema and infiltration of inflammatory cells.^3^, ^4^ Despite much research, the mortality rate is 36‐44%,^5^ and there is no effective therapeutic strategy.^6^

A connection between the lungs and gut has been demonstrated in human and animal studies.^7^, ^8^, ^9^, ^10^ A disturbance in the intestinal bacterial communities will disrupt the integrity of the intestinal barrier, thereby increasing the risk of bacterial translocation, activating systemic immune system and aggravating immune damage to the lung.^11^ For instance, stimulation of mouse lungs with lipopolysaccharide (LPS) causes acute changes in the structure and function of intestinal microflora, resulting in bacterial and endotoxin translocation and eventually lung injury, which is an important factor that initiates ARDS.^12^, ^13^, ^14^ Under physiological conditions, the intestinal mucosal barrier, gut microbiota and their metabolites together form the intestinal microecology, which maintains in a dynamic balance.^15^ However, the mechanism of how intestinal microflora changes affect lung injury during ARDS remains unclear. In addition, pneumonia induces intestinal injury and decreases the proliferation of gut mucosal epithelial cells.^16^ The mechanisms are likely related to the local inflammatory factor microenvironment and the immune response.^17^

Mesenchymal stem cells (MSCs), which are multipotent and immunoregulatory, have therapeutic potential in lung diseases.^18^ They can repair LPS‐induced ALI, pneumonia, inflammatory bowel disease (IBD) and systemic sepsis in animal models.^7^, ^19^ They protect against ALI by reducing inflammatory cytokine secretion, enhancing macrophage phagocytosis and regulating T‐cell differentiation.^20^, ^21^ Importantly, systemic administration of MSCs affects the gut epithelium and significantly ameliorates intestinal mucosal inflammation.^22^, ^23^, ^24^ The gut epithelium functions as a protective barrier against LPS under normal conditions. Small‐intestinal intraepithelial lymphocytes (IELs) are the first line of defence and are activated by inflammatory signals to eliminate infected epithelial cells.^25^ The CD8^+^ T cells infiltrating IELs produce interferon‐γ (IFN‐γ), which is correlated with the severity of intestinal damage.^26^ Moreover, the activation state of CD4^+^ T cells reflects the intestinal immune response.^27^ However, the mechanisms of MSC and IEL recruitment are unclear. Although MSCs have an immunomodulatory effect in ALI,^28^, ^29^ we propose that they not only have an immunomodulatory effect but also alter the intestinal microbiome in a manner that promotes barrier integrity.

In this study, we investigated the efficacy of MSCs in a mouse model of LPS‐induced ALI. Specifically, studies were done to investigate in greater detail the mechanisms by which MSCs may ameliorate the lung and intestinal injuries in ALI, including their effects on intestinal healing and faecal microbiome populations, as well as inflammation and immune response. The results indicate the important effects of MSCs on lung and gut healing by regulating the immune response of the lungs and intestines and gut microbiota, providing strong support for the use of MSCs as a uniform and sustainable source of cells for ALI therapy.

## 2 MATERIALS AND METHODS

### 2.1 Animals

Male wild‐type 6‐ to 8‐week‐old C57 BL/6J mice were purchased from Nanjing Biomedical Research Institute of Nanjing University, Nanjing, China. The same specific pathogen‐free room was used to house all mice. All animal experimental procedures were conducted according to a protocol approved by the Ethics Committee of The First Affiliated Hospital of Zhejiang University (No. 2015‐130).

### 2.2 Animal model of LPS‐induced acute lung injury

Lipopolysaccharide (derived from *Escherichia coli* 0111: B4, Sigma‐Aldrich, Poole, United Kingdom) was given intratracheally as a model of direct lung injury. The ALI model was induced by 20 mg/kg LPS as described previously,^30^ and negative control mice received an equal volume of phosphate‐buffered saline (PBS; PBS group; n = 10). Four hours after LPS administration, mice with ALI were randomly divided into the LPS/PBS (n = 40) and LPS/MSC (n = 40) groups. MSCs isolated from C57BL/6 mice compact bone were characterized by induced osteogenic and adipogenic differentiation (Supplemental Materials and Methods) and analysed the surface marker expression using flow cytometry (Figure S1). MSCs at passages 3‐5 were used in subsequent experiments. MSCs (5 × 10^5^, 20 μl total volume) were intratracheally instilled into mice in the LPS/MSC group; the mice in the LPS/PBS group received an equal volume of PBS, while mice in the negative control group were injected with PBS (PBS group; n = 10). Mice in the LPS/MSC group were randomly selected and anaesthetized at day 1 (M1d group, n = 8), day 3 (M3d group, n = 8) and day 7 (M7d group, n = 8). Likewise, mice were randomly selected from the LPS/PBS group and anaesthetized at day 1 (L1d group, n = 8), day 3 (L3d group, n = 8) and day 7 (L7d group, n = 8). Mortality was recorded every 24 hours until 12 days post‐transplantation. To deplete gut bacterial communities, mice were treated with broad‐spectrum antibiotics (ampicillin 1 g/L, Sangon Biotech, Shanghai, China; neomycin sulphate 1 g/L, Sangon Biotech; metronidazole 1 g/L, Sangon Biotech and vancomycin 0.5 g/L, Sangon Biotech) in drinking water for 3 weeks as described.^31^ Two days after cessation of the antibiotics, the mice were treated as described above.

### 2.3 Mouse tissue collection and processing

Mice were sacrificed for removal of the lungs, small‐intestinal segments and cecum and colon contents. Portions of the lung and intestinal tissues were fixed in 10% neutral buffered formalin to prepare paraffin‐embedded sections, and sliced into 5‐μm‐thick sections. Another set of mice was sacrificed. The lungs were washed three times with 800 µl ice‐cold PBS through a tracheal cannula as described previously,^32^ and the bronchoalveolar lavage fluid (BALF) was collected and centrifuged at 800 g for 10 min at 4°C. The supernatant was stored at −80°C for subsequent assay of cytokines and protein concentration, and the cell pellet was resuspended in 200 μl for cell counting and cell smear generation. The total protein content of BALF was quantified using the Pierce™ BCA Protein Assay Kit (Thermo Fisher Scientific, Inc).

### 2.4 Cytokine analyses

BALF and small‐intestinal tissue homogenate supernatants were harvested and analysed for their contents of tumour necrosis factor‐α (TNF‐α), interferon‐γ (IFN‐γ), interleukin‐6 (IL‐6), IL‐1α and IL‐1β by bead‐based multiplex LEGENDplex™ assay (Multi‐Analyte Flow Assay Kit, BioLegend, Koblenz, Germany) according to the manufacturer's instructions.

### 2.5 Lung and intestinal histology

For histopathologic analyses, formalin‐fixed lung and ileum samples were paraffin‐embedded, sectioned (5 μm thickness) and stained with haematoxylin and eosin (H&E). The sections were evaluated by an experienced pathologist who was blinded to the group assignment. Images were scanned using a NanoZoomer 2.0‐RS scanner (Hamamatsu Photonics KK, Hamamatsu, Shizuoka, Japan) captured at a magnification of 20 × or 40 × using NDP.view.2 software (Hamamatsu Photonics KK).

### 2.6 Isolation of pulmonary immune cells, IELs and PPs

Lung tissues were harvested, cut into 1 cm pieces, transferred to a gentleMACS C tube (Miltenyi Biotec, Bergisch Gladbach, Germany) containing an enzyme mixture (Mouse Lung Dissociation Kit, Miltenyi Biotec), homogenized and digested at 37°C for 30 minute. Pulmonary immune cells were obtained after density gradient centrifugation.

Peyer's patch (PP) single‐cell suspensions were prepared by mashing the organs through 70‐μm cell strainers (JET Biofil, Guangzhou, China). Small‐intestinal intraepithelial lymphocytes (IELs) were isolated as described previously.^33^ Briefly, PPs were removed, and the small intestine was opened longitudinally and washed three times with Roswell Park Memorial Institute (RPMI) 1640 (Gibco, CA) containing 10% foetal bovine serum (FBS; Gibco), penicillin (100 U/ml) and streptomycin (100 μg/ml). Next, intestinal tissues were shaken in RPMI 1640 medium containing 5 mM ethylenediaminetetraacetic acid (Sangon Biotech) and 5% FBS at 175 rpm and 37°C for 15 min. This step was repeated four times, and the supernatants were discarded. Pelleted cells were directly suspended in 5 ml 40% Percoll and overlaid onto 3 ml 70% Percoll (GE Healthcare, Uppsala, Sweden); the cells layering between the 40% and 70% fractions were collected as IELs.

### 2.7 Mass cytometry antibody staining

Mass cytometry was used to evaluate the CD8^+^ T‐cell populations in mouse lung. Pulmonary immune cell suspensions were washed once in 1 ml staining buffer (PBS with 0.5% BSA and 0.02% NaN_3_) and incubated with 0.25 μM cisplatin (Fluidigm, San Francisco, CA) on ice for 5 minute to label dead cells. Next, the cells were labelled with a mixture of metal isotope‐conjugated antibodies against cell surface markers for 30 minute on ice. Then, 0.03 μM Ir nucleic‐acid intercalator (Fluidigm) in Fix and Perm Buffer was added, and the cell suspensions were placed at 4°C overnight. The cells were washed twice and incubated with a metal isotope‐conjugated intracellular antibody cocktail for 30 minute on ice. The cells were resuspended in distilled water containing 20% EQ. 4 beads (Fluidigm) and acquired on a CyTOF instrument with a Helios system (Fluidigm) at 500 events per second.

### 2.8 Flow cytometry

The single‐cell suspensions of IELs and PPs were stained using standard procedures for cell surface markers. The following monoclonal antibodies were used for flow cytometry: FITC‐CD45 (30‐F11; BD Biosciences, New Jersey, USA), BV395‐CD3e (145‐2C11; BD), PECy7‐TCRγδ (GL3; BioLegend), BV605‐CD4 (RM4‐5; BD), PerCPcy5.5‐CD8α (53‐6.7; BD), APC‐CD11b (M1/70; BD) and PE‐F4/80 (T45‐2342). Dead cells were excluded using Fixable Viability Stain 510 (BD). Flow cytometry analyses were performed using an LSR Fortessa (BD).

### 2.9 DNA extraction

Following homogenization of the mouse cecum and colon contents using glass beads in a Precellys 24 homogenizer (Bertin Technologies, Montigny, France), bacterial DNA was extracted using a QIAamp DNA Stool Mini Kit (Qiagen) according to the manufacturer's protocol. DNA extracts were stored at –80°C until use.

### 2.10 16S rRNA gene amplification and DNA sequencing

Bacterial community diversity was analysed via single‐molecule real‐time PacBio sequencing (Pacific Biosciences, Menlo Park, CA).^34^, ^35^ The full‐length 16S ribosomal RNA gene was amplified from genomic DNA using the bacteria‐specific primers 27 F (5′‐AGAGTTTGATCMTGGCTCAG) and 1492 R (5′‐TACGGYTACCTTGTTACGACTT). The quality and concentration of the PCR products were determined via electrophoresis on 2% agarose gels. Sequencing libraries were constructed using the SMRTbell Template Prep Kit 1.0‐SPv3 according to the manufacturer's instructions (Pacific Biosciences).

No sequences contained ambiguous bases. Details of the sequencing output, such as raw reads, clean reads and average read lengths, are summarized in supplemental Table S1. Operational taxonomic unit (OTU) clustering was performed using a 99% similarity cut‐off to represent one species per OTU. After sequencing data were rarefied, the alpha and beta diversities were calculated, and differences in relative abundance were compared using Statistical Analyses of Metagenomic Profiles (STAMP) software.

### 2.11 Statistical analyses

We used the Kaplan–Meier log‐rank test to compare survival between groups (GraphPad Prism 8). Two‐tailed, unpaired Student's t‐tests were performed for comparing two groups. Quantification of immunohistochemical assays was performed using National Institutes of Health image analysis software ImageJ bundled with 64‐bit Java 1.8.0_172. The alpha diversity, including the indexes of Shannon, Chao1 and observed species, was calculated using QIIME (Version 1.9.1). Unweighted uniFrac principal coordinates analysis and weighted uniFrac principal coordinates analysis (PCoA) were performed after rarefaction and log standardization of OTU table using R version 2.15.3 and the package stats and ggplot2. Analysis of similarities (ANOSIM) was used to evaluate differences in overall microbiota composition between the groups using weighted and unweighted UniFrac distance matrices. Linear discriminant analysis (LDA) effect size was utilized to evaluate differentially abundant bacterial taxa and predicted function between the animal groups. Data are presented as means ± standard error of the mean (SEM). We set three levels of statistical significance (*P <.05; **P <.01; ***P <.001).

## 3 RESULTS

### 3.1 MSCs improve survival and attenuate lung injury in LPS‐induced ALI mice

**FIGURE 1:**
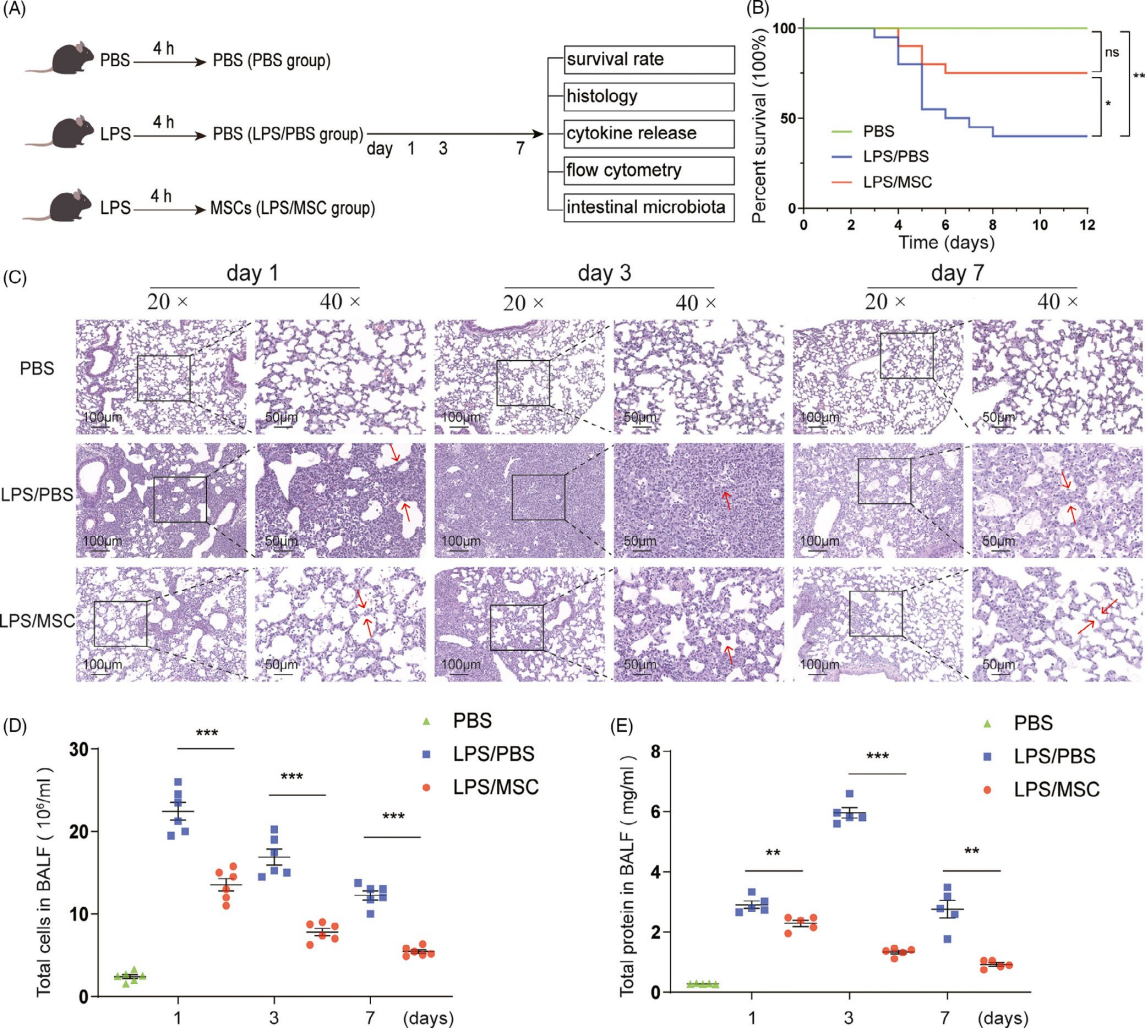
Mesenchymal stem cells (MSCs) alleviate lipopolysaccharide (LPS)‐induced lung injury (ALI). (A) Schematic showing the three time points of ALI after intratracheal instillation of LPS. (B) Kaplan–Meier survival curves of LPS‐induced ALI mice after intratracheal delivery of 0.5 × 10^6^ MSCs or phosphate‐buffered saline (PBS) for 12 days. Green line, C57/BL6 mice treated with PBS (PBS group, n = 10); blue line, C57/BL6 mice induced by LPS with PBS (LPS/PBS group, n = 20); red line, LPS‐induced mice treated with MSCs (LPS/MSC group, n = 20). *P <.05, **P <.01, ns, not significant. (C) Haematoxylin–eosin (HE) staining of lung sections of C57/BL6 mice at the indicated times after LPS injection. Magnifications × 20 (scale bar = 100 μm) and × 40 (scale bar = 50 μm). On days 1 and 3 after LPS treatment, many immune cells infiltrated the alveolar interstitium, which was thickened; by contrast, MSCs treatment reduced immune cell infiltration and alveolar interstitium thickness. (D) Total cell count and (E) total protein concentration in bronchoalveolar lavage fluid (BALF) of LPS‐induced ALI mice with or without MSC treatment at days 1, 3 and 7. BALF total cell count peaked in the LPS/PBS group at day 1 and decreased at day 7 and was lower in the LPS/MSC group. The BALF protein concentration peaked at day 3 and decreased at day 7 in the LPS/PBS group and was markedly lower in the LPS/MSC group (mean ± SEM, n = 5 or 6). **P <.01, ***P <.001 by Student's t‐test

We constructed a stable ALI mouse model via intratracheal injection of 20 mg/kg LPS. Mouse MSCs were characterized as shown in Figure S1. The ALI mice were administered LPS; after 4 hours, MSCs or PBS was given orotracheally (Figure 1A). To determine how MSCs ameliorate LPS‐induced ALI, mice were divided into three groups: PBS, LPS/PBS and LPS/MSC groups. Twelve days after LPS exposure, the survival rate in the LPS/MSC group was significantly greater than that in the LPS/PBS group (75% vs. 40%; *P* <.05) (Figure 1B). The degree of lung injury was assessed based on the lung histology (Figure 1C), BALF total cell count (Figure 1D) and BALF protein concentration (Figure 1E). Diffuse alveolar and interstitial infiltration and thickening of the alveolar walls and interstitium were evident in the LPS/PBS group. By contrast, the alveolar walls and interstitium were thinner and immune cell infiltration was reduced in the LPS/MSC group (Figure 1C). Total cell count and total protein concentration in BALF were significantly reduced, by MSCs, confirming that these cells attenuate lung injury in LPS‐induced ALI mice (Figure 1D and 1E).

### 3.2 MSCs reduce intestinal mucosal injury and improve intestinal barrier integrity

**FIGURE 2:**
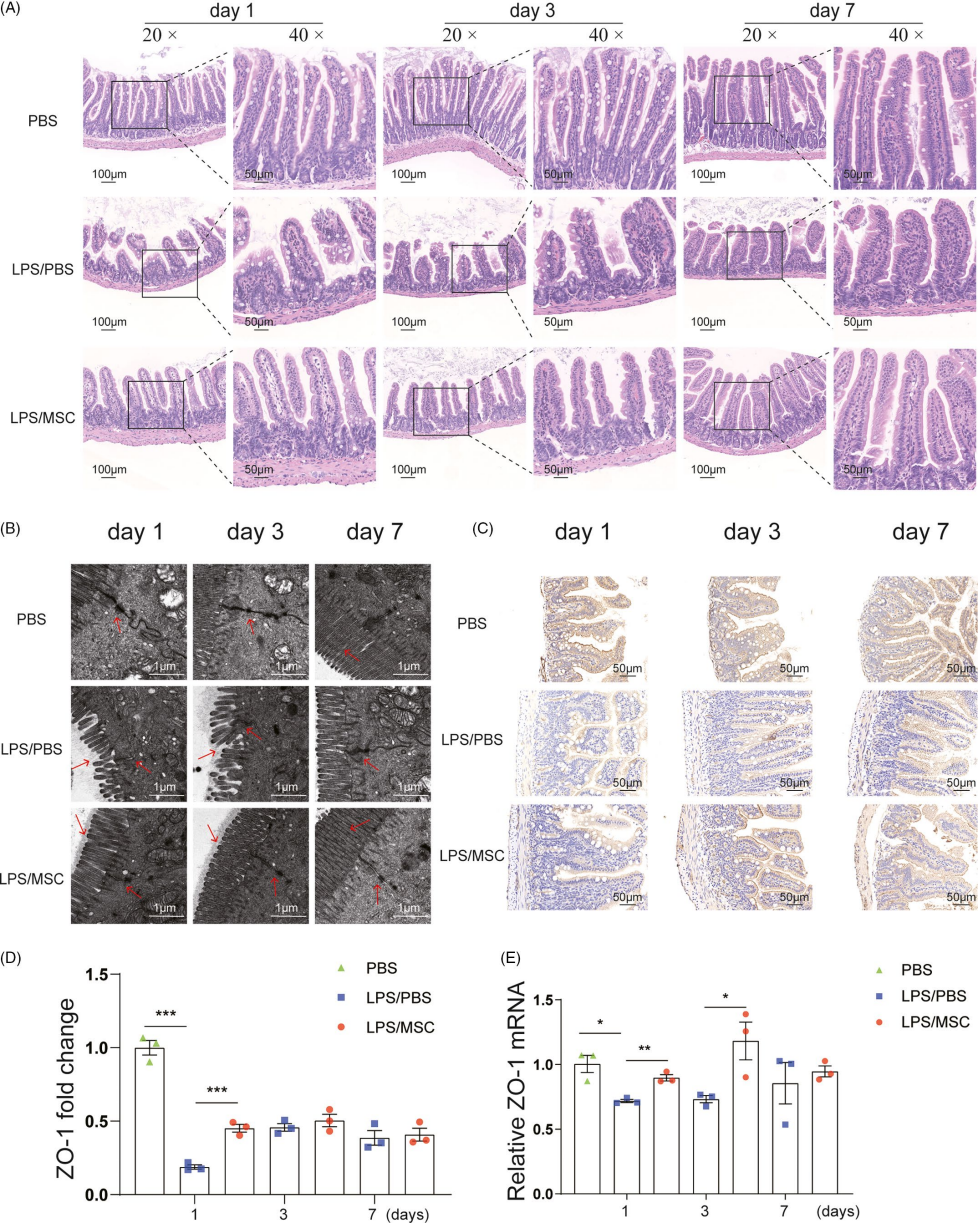
MSCs improved intestinal barrier function. (A) Representative images of histological changes in the ileum assessed by H&E staining. Magnifications × 20 (scale bar = 100 μm) and × 40 (scale bar = 50 μm). The intestinal villi shrank and ruptured in the LPS/PBS group at days 1 and 3, and ileal mucosal tissues demonstrated reversion to villi of normal length and muscular layers of typical architecture in the LPS/MSC group. (B) Ileal mucosal ultrastructure of LPS‐induced ALI mice with or without MSC treatment. Magnification × 30 000 (scale bar = 1 μm). The microvilli of intestinal epithelial cells were sparse and shorter in the LPS/PBS group at days 1 and 3, and these changes were substantially ameliorated in the LPS/MSC group. (C) Ileal ZO‐1 immunohistochemical staining revealed increased positive signals in intestinal tissue at day 1 in the LPS/MSC group than the LPS/PBS group. Magnification × 40 (scale bar = 50 μm). (D) Semiquantitative determination of ZO‐1 positive signals using ImageJ (mean ± SEM, n = 3). ***P <.001 by Student's t‐test. (E) Ileal ZO‐1 expression was determined by quantitative qPCR. Relative RNA levels were calculated by the ΔΔCt method using β‐actin for normalization (mean ± SEM, n = 3). *P <.05, **P <.01 by Student's t‐test

Stimulation of mouse lungs with LPS has been shown to alter microbial diversity and abundance, which plays a vital role in preventing the invasion of pathogenic bacteria and maintaining the immune homeostasis of the gut mucosa as a microbial barrier,^16^, ^36^, ^37^ and therefore, we investigated intestinal barrier integrity in this study. Histological inspection of ileal mucosal tissues in the LPS/PBS group demonstrated villus atrophy and breakage, and intestinal epithelial destruction. In contrast, MSCs restored a normal villus length and muscular‐layer architecture (Figure 2A). The microvilli of intestinal epithelial cells in the LPS/PBS group were sparse and shortened at day 1, and these changes were substantially ameliorated in the LPS/MSC group (Figure 2B). We assayed the expression of the tight junction protein ZO‐1 by immunohistochemistry staining and qPCR. Immunohistochemistry staining showed increased ZO‐1 expression on the ileal membrane in the LPS/MSC group at day 1 (Figure 2C and 2D). At days 1 and 3, the ZO‐1 mRNA level in the LPS/MSC group was higher than that in the LPS/PBS group (Figure 2E). At day 7, the structure of the gut gradually returned to that of normal in both LPS/PBS and LPS/MSC groups. Therefore, MSCs protected the intestinal epithelial barrier in LPS‐induced ALI mice in the early stages of the disease.

### 3.3 MSCs attenuate lung and intestinal inflammation caused by LPS

**FIGURE 3:**
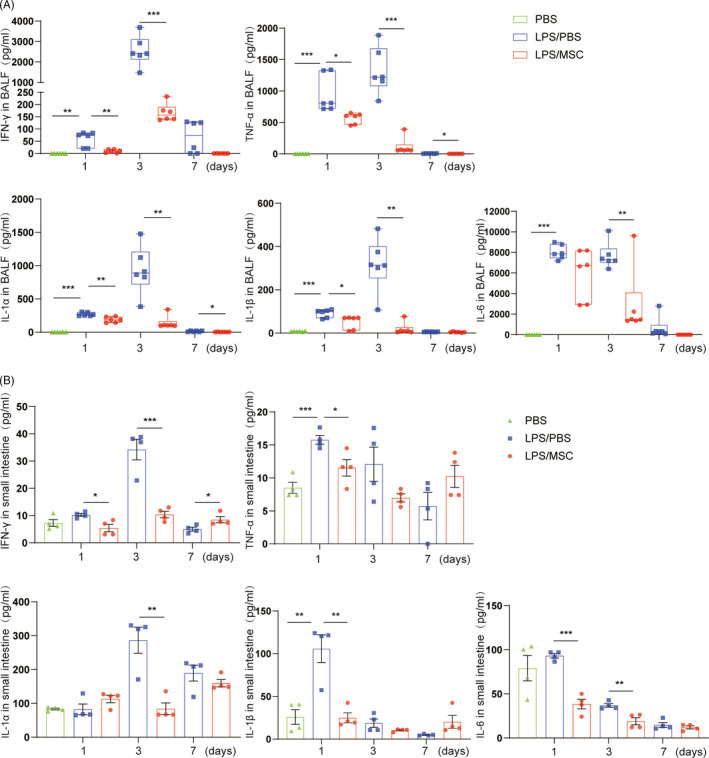
Therapeutic effects of MSCs on cytokines in the lung and gut in LPS‐induced acute lung injury. The IFN‐γ, TNF‐α, IL‐1α, IL‐1β and IL‐6 concentrations were determined at days 1, 3 and 7 in the PBS, LPS/PBS, LPS/MSC groups by bead‐based multiplex LEGENDplex™ assay. (A) Inflammatory cytokine concentrations in BALF were significantly increased in the LPS/PBS group, but were lower in the LPS/MSC group. MSCs decreased the cytokine concentrations in BALF (mean ± SEM, n = 6). (B) Cytokine levels in the small intestine increased, particularly at days 1 and 3, in the LPS/PBS group compared with the PBS group. Administration of MSCs reduced the LPS‐induced increased cytokine concentrations in intestinal tissue (mean ± SEM, n = 4). *P <.05, **P <.01, ***P <.001 by Student's t‐test

We assayed the levels of IFN‐γ, TNF‐α, IL‐1α, IL‐1β and IL‐6 in the lung and small intestine. LPS induced lung inflammation, as evidenced by increased IFN‐γ, TNF‐α, IL‐1α, IL‐1β and IL‐6 levels in BALF at days 1 and 3 compared with the PBS group (Figure 3A). LPS also induced small‐intestinal inflammation: IFN‐γ, TNF‐α, IL‐1α, IL‐1β and IL‐6 levels in the small intestine were increased at days 1 and 3 (Figure 3B), suggesting that LPS‐induced lung injury resulted in low‐grade gut inflammation. MSCs significantly alleviated the LPS‐induced inflammatory response in BALF. Similarly, intestinal inflammatory cytokine levels were downregulated to almost the level of the control in the LPS/MSC group. Therefore, MSCs attenuated tissue injury by inhibiting increased expression of cytokines in the lung and gut.

### 3.4 Effects of MSC administration on lung and gut immunoregulation

**FIGURE 4:**
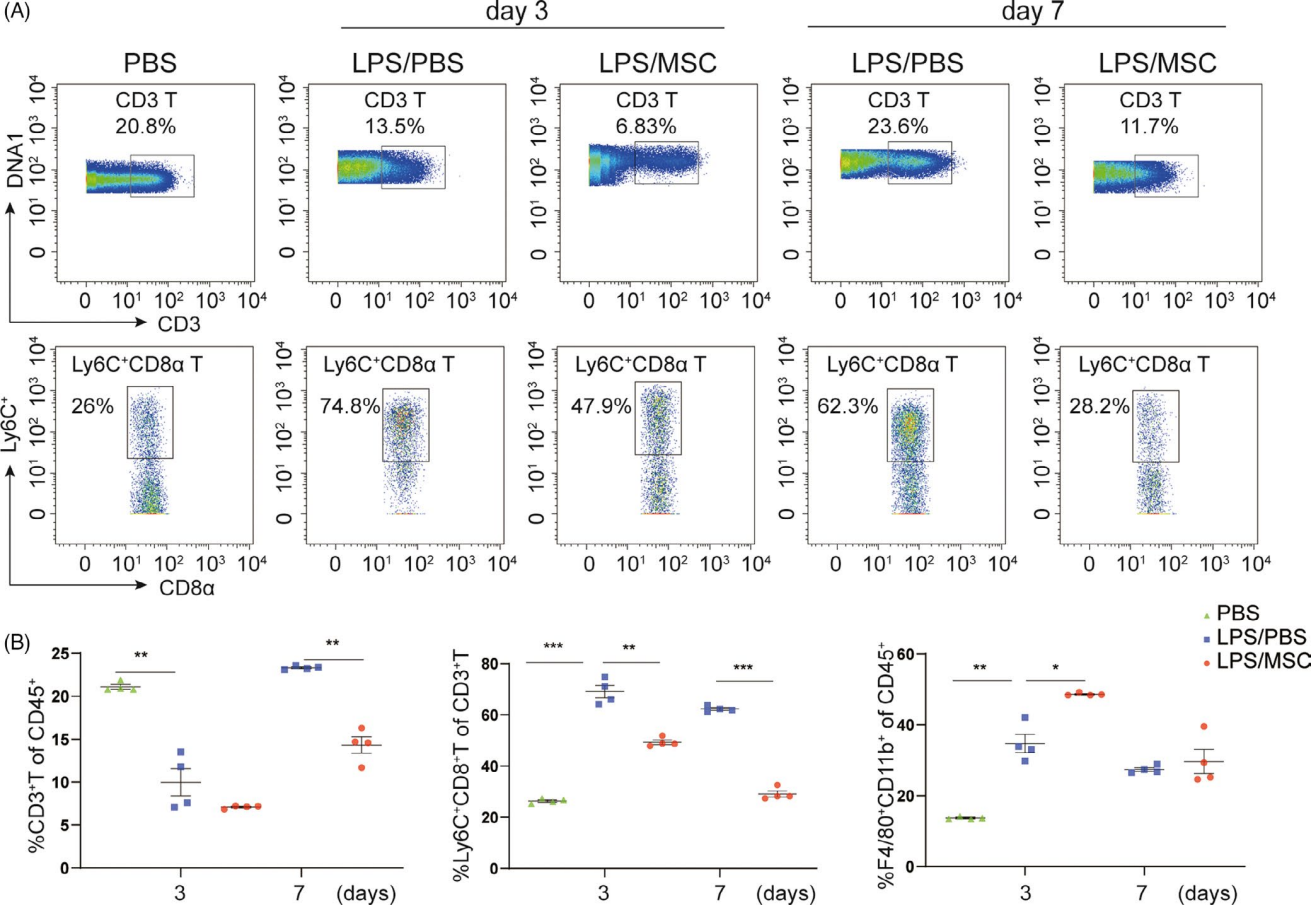
Immunomodulatory effects of MSCs in the lung. (A) Mass cytometry analyses of CD3^+^ and Ly6C^+^ CD8^+^ T‐cell subsets in a representative mouse. Numbers are the fractions of cells in the indicated gate. (B) Frequency of CD45^+^ immune cells in lung tissue at days 3 and 7 in the PBS, LPS/PBS and LPS/MSC groups. The proportion of Ly6C^+^CD8^+^ T cells was markedly lower in the LPS/MSC group than in the LPS/PBS group. Each symbol indicates an individual mouse (mean ± SEM, n = 4). *P <.05, **P <.01, ***P <.001 by Student's t‐test

To determine the effects of MSCs on immune responses in the lung and small intestine in ALI, we analysed CD45^+^ (lymphocyte common antigen) cells in LPS‐treated mice. CD8^+^ T cells in the lungs were analysed by mass cytometry using the reasonable gating strategy. The populations of CD3^+^ T cells and Ly6C^+^CD8^+^ T cells in lung immune compartments were significantly increased in the LPS/PBS group compared with the PBS group (Figure 4A). In mice treated with MSCs, the populations of CD3^+^ T cells and Ly6C^+^CD8^+^ T cells were significantly reduced compared with LPS‐treated mice at day 7, and F4/80^+^CD11b^+^ macrophages were recruited to the injured lung in the LPS/MSC group at day 3 (Figure 4B). Therefore, MSCs regulate systemic immunity by modulating the CD8^+^ T‐cell response and macrophage recruitment to the injured lung.

**FIGURE 5:**
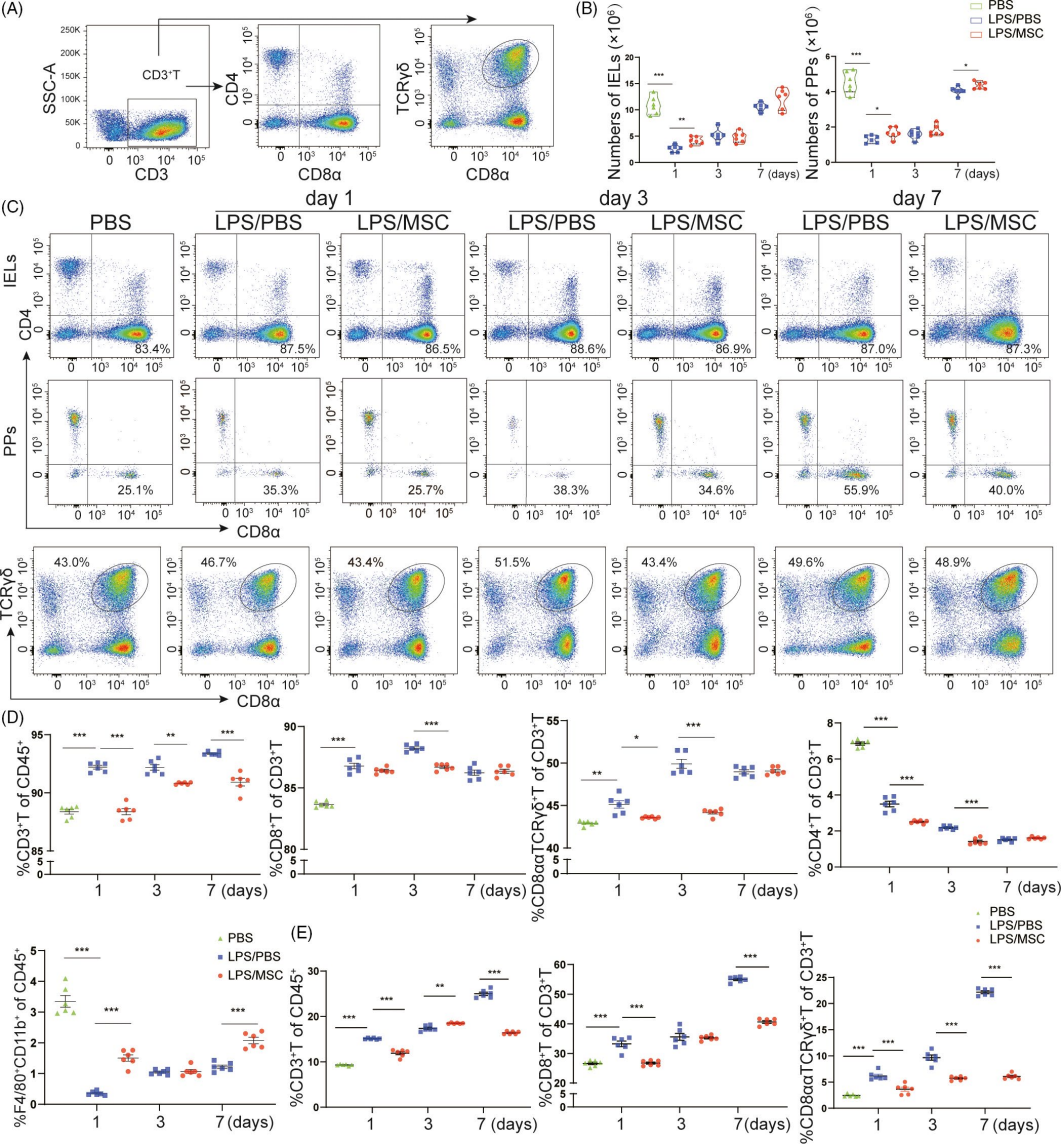
Immunomodulatory effects of MSCs in the intestine. (A) Density plots of the gating strategy for CD8^+^ T cells among small‐intestinal intraepithelial lymphocytes (IELs) and Peyer's patches (PPs) of ALI mice by flow cytometry. (B) Total numbers of IELs and PPs in mice with acute lung injury mice. Each symbol indicates an individual mouse (mean ± SEM, n = 6). (C) Flow cytometric analyses of CD8^+^T cells among IELs and PPs from the indicated mice. (D) Frequency of CD3^+^ T, CD4^+^ T, CD8^+^ T cells and F4/80^+^CD11b^+^ cells in IELs in the PBS, LPS/PBS and LPS/MSC groups. A decrease in the percentage of CD8ααTCRγδ^+^ T cells was observed in the LPS/MSC group at days 1 and 3. Each symbol indicates an individual mouse (mean ± SEM, n = 6). *P <.05, **P <.01, ***P <.001 by Student's t‐test. (E) The frequency of T cells in PPs at days 1, 3 and 7 in PBS, LPS/PBS and LPS/MSC groups. Each symbol indicates an individual mouse. (mean ± SEM, n = 6). **P <.01, ***P <.001 by Student's t‐test

Next, we evaluated the numbers and phenotypes of CD45^+^ cells in small‐intestinal IELs and PPs (Figure 5A). Compared to the PBS group, the numbers of IELs and PPs were decreased in the LPS/PBS group and increased in the LPS/MSC group at day 1 (Figure 5B). In addition, the population of CD8^+^ T cells in IELs was drastically increased, but that of CD4^+^ T and F4/80^+^CD11b^+^ macrophages was decreased, in the LPS/PBS group compared with the PBS group (Figure 5C and 5D). MSCs restored the CD8^+^ T‐cell–macrophage balance in ALI mice. By contrast, the changes in the CD8^+^ T‐cell populations in PPs were consistent with those in the intestinal epithelium (Figure 5E). Next, we sought to establish whether MSCs influenced T‐cell receptor (TCR) γδ^+^ T‐cell recruitment in IELs and PPs. TCRγδ^+^ T cells were mostly expressed in intestinal epithelial cells and expressed at a low level in PPs in PBS group (Figure S2). However, unexpectedly, their levels were markedly increased in IELs in the LPS/PBS group at day 3, as were CD8ααTCRγδ^+^ T levels (Figure 5C and 5D). By contrast, at day 3 the population of TCRγδ^+^ T cells in IELs was reduced, as was that of CD8ααTCRγδ^+^ T cells, in the LPS/MSC group, suggesting that MSCs ameliorated intestinal T‐cell infiltration. Therefore, MSCs reduced intestinal epithelial inflammation by regulating the immune cell populations in lung and gut tissues.

### 3.5 Gut bacterial community composition is altered by MSCs

**FIGURE 6:**
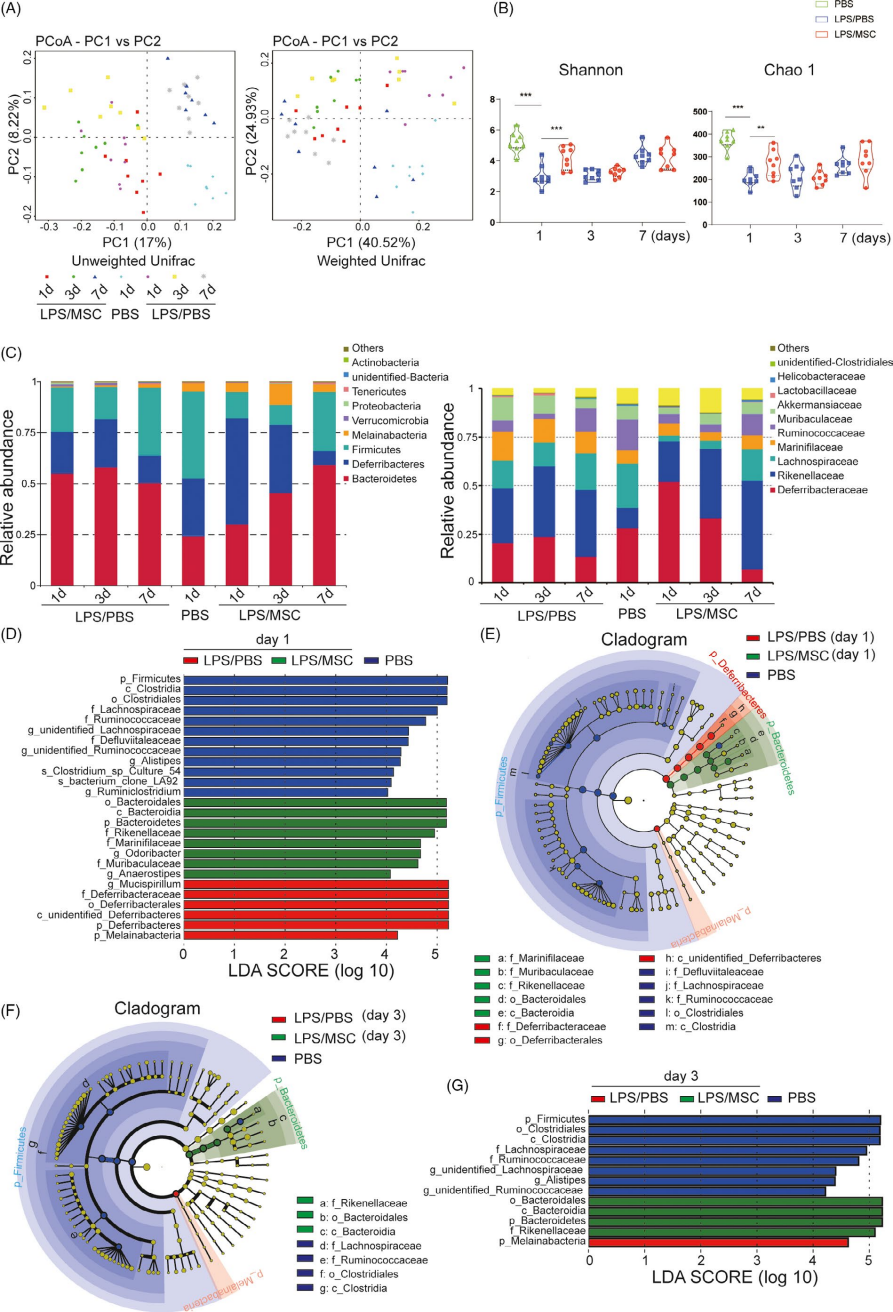
Intestinal bacterial community composition was altered following lung injury. (A) Analysis of the variance between microbial communities in the faeces of mice assessed by average relative abundance using principal coordinates analysis (PCoA) and full‐length 16S rRNA sequencing. PCoA plots and principal coordinates (PC) 1 and PC 2. Differences are expressed as unweighted and weighted UniFrac distances, respectively. Each symbol indicates an individual mouse (n = 8). (B) α‐Diversity of 16 S rRNA gene sequences at days 1, 3 and 7 in the PBS, LPS/PBS and LPS/MSC groups. Each symbol indicates an individual mouse (mean ± SEM, n = 8). **P <.01, ***P <.001 by Student's t‐test. (C) Average relative abundance of microbiota at the phylum and family levels in the PBS, LPS/PBS and LPS/MSC groups. (D, E) Linear discriminant analysis (LDA) effect size (LEfSe) based on 16 S rRNA gene sequences at day 1 and (F, G) day 3 (α = 0.01, LDA score >4.0). Histogram of the LDA scores for differentially abundant bacterial taxa in the PBS, LPS/PBS and LPS/MSC groups (n = 8)

Lipopolysaccharide‐induced lung injury has been shown to alter gut bacterial communities.^12^ Thus, we analysed the effects of MSCs on intestinal microbial communities by sequencing full‐length 16S rDNA amplicons from 56 faecal samples from ALI mice. Rarefaction curves for the observed species in all samples approached a plateau, indicating that the PacBio sequencing covered all OTUs (Figure S3). To characterize the global differences in microbial communities between groups, principal coordinate analysis (PCoA) was performed on unweighted UniFrac distances and weighted UniFrac distances. The PCoA plots showed significant separation at days 1 and 3, but not at day 7, between the LPS/PBS and LPS/MSC groups (Figure 6A), based on weighted UniFrac distances. Furthermore, ANOSIM revealed a significant difference in beta diversity among the groups (Figure S4A). At day 1, the number of species was decreased in the LPS/PBS compared with the PBS group (Figure S4B). In addition, the Shannon and Chao1 indices suggested a significantly decreased diversity in the LPS/PBS group (Figure 6B). In addition, the MSC‐treated groups shared more OTUs with the control group than the LPS/PBS group at day 1 (Figure S4C).

The dominant phyla in all mice were Bacteroidetes, Deferribacteres and Firmicutes (~88‐97% combined total relative abundance). Other phyla detected included Melainabacteria, Verrucomicrobia and Proteobacteria (Figure 6C). At the family level, there were trends of differential abundance in members of the family Lachnospiraceae (phylum Firmicutes) and certain members of the phyla Bacteroidetes and Deferribacteres at different time points. Notably, the abundance of the family Deferribacteraceae increased at day 1 after LPS exposure, whereas that of the family Lachnospiraceae increased in MSC‐treated mice. Linear discriminant analysis (LDA) effect size (LEfSe) analyses were conducted to identify differentially abundant bacterial taxa (Figure 6D–6G). In all, 26 bacterial groups were differentially abundant (α = 0.01, LDA score >4.0) between the PBS, LPS/PBS and LPS/MSC groups at day 1 (Figure 6D). LPS treatment resulted in significantly decreased relative frequencies of the families Lachnospiraceae, Ruminococcaceae and Defluviitaleaceae (class Clostridia; phylum Firmicutes) in the LPS/PBS group compared with the PBS group (Figure 6E). At day 3, the abundance of several genera, mostly the class Bacteroidia, was increased in the LPS/MSC group compared with the LPS/PBS group (Figure 6F and 6G). The results of LEfSe analyses of the LPS/PBS and LPS/MSC groups at days 1, 3 and 7 are shown in Figure S4D. Together, the findings indicate that MSCs restored a healthy intestinal bacterial community composition.

### 3.6 The impact of gut microbiota depletion on the efficacy of MSCs in ALI mice

**FIGURE 7:**
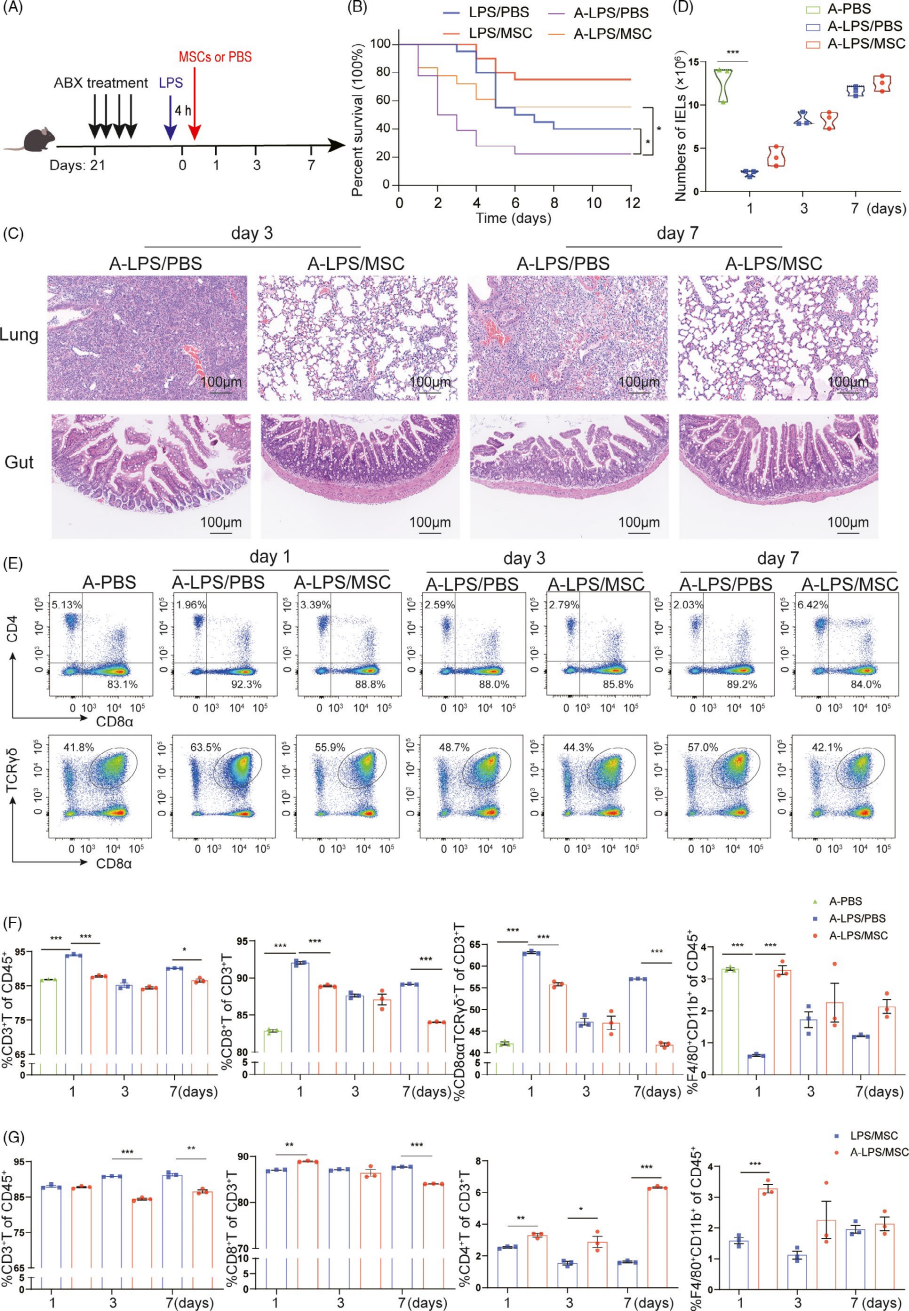
Effects of MSCs on antibiotic‐treated ALI mice. (A) Treatment with four broad‐spectrum antibiotics (ABX) in drinking water for 3 weeks before LPS injection in C57/BL mice to deplete the gut microbiota. Also shown is the treatment schedule for ABX water feeding, LPS injection and MSCs or PBS intratracheal instillation. (B) Kaplan–Meier survival curves of LPS‐induced ALI mice after ABX treatment. Blue line and red line are as shown in Figure 1B; purple line, C57/BL6 mice induced by LPS without MSCs (A‐LPS/PBS group, n = 18); orange line, LPS‐induced mice treated with MSCs (A‐LPS/MSC group, n = 18). *P <.05. (C) Representative images of histological changes in the ileum and lung assessed by H&E staining. Magnifications 20× (scale bar = 100 μm). On days 3 and 7 after LPS treatment, the lung of the A‐LPS/PBS group exhibited a more severely distorted structure. A large number of inflammatory cell infiltrations were observed in the pulmonary interstitium, and many red blood cells were observed in the lung vasculature, while leakage occurred in the bronchial lumen. A‐LPS/PBS group also showed gut villus separation and gut mucosal damage. (D) Numbers of IELs over time in antibiotic‐treated ALI mice with or without MSCs transplantation. Each symbol indicates an individual mouse (mean ± SEM, n = 3). (E) Representative flow cytometry plots of CD8^+^ T cells in IELs showing the effects of MSCs on antibiotic‐treated mice at days 1 and 7. (F) Flow cytometry analyses of T cells and macrophages in IELs of ALI mice in the A‐PBS, A‐LPS/PBS and A‐LPS/MSC groups. Each symbol indicates an individual mouse (mean ± SEM, n = 3). (G) Flow cytometry results of the LPS/MSC and A‐LPS/MSC groups at three time points (mean ± SEM, n = 3). *P <.05, **P <.01, ***P <.001 by Student's t‐test. ABX/A: mice treated with antibiotics

The gut bacterial community composition is associated with peripheral immunity homeostasis.^38^, ^39^ Therefore, bacterial community dysbiosis may modulate the therapeutic effects of MCSs. We treated C57/BL mice with four broad‐spectrum antibiotics (ampicillin, neomycin, metronidazole and vancomycin) (ABX) in drinking water for 3 weeks before LPS injection to deplete the gut microbiota (Figure 7A). Subsequently, mice received a single dose of LPS (20 mg/kg) to induce ALI. After 4 hours, half of the LPS‐treated mice received MSCs. Mice in the negative control group were injected with PBS after ABX treatment. Mice were assigned to the A‐PBS (n = 6), A‐LPS/PBS (n = 30) and A‐LPS/MSC (n = 30) groups. The survival rate in the A‐LPS/MSC group was significantly greater than in the A‐LPS/PBS group (56% vs. 22%, *P* <.05) (Figure 7B), indicating that lung injury was severe during ALI in antibiotic‐treated mice and was significantly alleviated after administration of MSCs. Moreover, the survival curve also showed that, compared to the LPS/PBS group, the A‐LPS/PBS group had a higher mortality rate (78% vs. 60%, *P* <.05). Antibiotic administration altered the gut mechanical barrier in mice, which resulted in gut villus separation and gut mucosal damage (Figure 7C). These changes were evident in the A‐LPS/PBS group compared with the LPS/PBS group. The histopathological changes also showed the same trend in the lung; that is, compared to the LPS/PBS group, the lung of the A‐LPS/PBS group exhibited a more severely distorted structure (Figure 7C). A large number of inflammatory cell infiltrations were observed in the pulmonary interstitium, and many red blood cells were observed in the lung vasculature, while leakage occurred in the bronchial lumen at day 3 and day 7 in the A‐LPS/PBS group.

The number of IELs decreased significantly at day 1 in the A‐LPS/PBS group and was similar to those in the other groups (Figure 7D). The CD3^+^ T‐cell population, in particular CD8^+^ T, CD8ααTCRγδ^+^ T cells, was dramatically altered (Figure 7E). In addition, CD8^+^ T‐cell populations among IELs were significantly increased, and those of F4/80^+^CD11b^+^ macrophages were reduced, in the A‐LPS/PBS group. The percentages of CD8^+^ T cells and CD8ααTCRγδ^+^ T cells were lower in the A‐LPS/MSC group, similar to mice that were not treated with antibiotics (Figure 7F).

Next, we analysed IELs from the LPS/MSC and A‐LPS/MSC groups at three time points. The effect of MSCs on T‐cell populations and macrophages was more apparent in antibiotic‐treated mice compared with MSCs alone (Figure 7G). Therefore, the immunomodulatory effects of MSCs on ALI were mediated by changes in bacterial community composition in the gut.

## 4 DISCUSSION

There is substantial evidence on the immunomodulatory effects of MSCs in ALI,^40^ which is mediated via amelioration of lung inflammation and immunosuppression.^41^ However, although crosstalk between the gut and lung occurs in pulmonary diseases,^42^, ^43^ the gut–lung axis is not fully understood. Moreover, the means by which MSCs regulate intestinal immunity and their relationship with intestinal bacterial communities are unclear. Therefore, we assessed the protective effects of MSCs in the lung and gut of LPS‐induced ALI mice. The survival rate and histopathology analyses indicated that tissue injury was severe at day 1 and day 3 after ALI; the tissue gradually returned to normal by day 7, and MSC treatment improved the survival of ALI mice. Days 1, 3 and 7 were therefore chosen to represent the initial inflammatory phase and resolution phase, respectively. The data suggest that MSCs enhanced survival, attenuated lung and gut inflammation, improved intestinal barrier function and reduced intestinal infiltration of CD8^+^ T cells in ALI mice with or without antibiotic treatment during the initial inflammatory phase. Further, depletion of intestinal bacterial communities with antibiotics could thus contribute to development or severity of the pulmonary disease as evidenced by the increased lung and gut damages and mortality (Figure 7).

The gut barrier mainly includes a mechanical barrier, a microbial barrier, a chemical barrier and an immune barrier.^44^ The mechanical barrier is the first line of defence against infection, which mainly includes the gut epithelial cell layer and tight junctions.^45^ The mucus layer has an important role in inhibiting the invasion and adhesion of pathogenic bacteria to epithelial cells.^46^ Various gut microbiota also play an essential role in preventing the invasion of pathogenic bacteria and maintaining the immune homeostasis of the gut mucosa.^36^ In our study, histological examination, 16S analysis and flow cytometry results showed that LPS‐induced ALI damaged the intestinal barrier, while the application of MSCs helped prevent or ameliorate ALI.

An unresolved issue is how LPS affects intestinal inflammation and injury. In this study, LPS‐induced ALI mice were in a classic pulmonary inflammatory state during ALI progression. However, survival, lung pathological lesions and inflammatory cells and total protein in BALF were markedly decreased by MSCs. Interestingly, histological examination of the ileum revealed a mild lesion characterized by an impaired intestinal barrier at days 1 and 3 after LPS exposure. The ALI mice with an imbalance in the gut microbiota showed more obvious gut damage and increased mortality; in contrast, MSCs were shown to reduce gut damage (Figure 7). Therefore, MSCs enhanced the barrier function of the intestinal mucosa.

The anti‐inflammatory properties of MSCs have been described.^32^, ^47^ In this study, MSCs ameliorated inflammatory response in BALF, as indicated by decreased levels of IFN‐γ, TNF‐α, IL‐1α, IL‐1β and IL‐6. Our results are consistent with the previous studies on the anti‐inflammatory effects of MSCs in ALI.^20^, ^48^ Similarly, cytokine levels in ileal tissues were increased in the LPS/PBS group, suggesting that LPS‐induced lung injury results in low‐grade gut inflammation, and MSCs significantly ameliorated these increases. Kusaka et al reported that Ly6C^+^CD8^+^ T cells produce IFN‐γ during the acute phase of infection.^49^ The downregulation of inflammatory cytokine expression may be related to the immunosuppressive effects of MSCs.

Modulation of the mucosal immune response shows promise for alleviating the pulmonary and intestinal inflammatory injury induced by LPS. The immunomodulatory effects of MSCs in the respiratory system involve T‐cell differentiation.^32^ In this study, the therapeutic effects of MSCs were found to be mediated by reduced infiltration of Ly6C^+^CD8^+^ T cells, in line with the lower IFN‐γ level in the LPS/MSC group, since excessive IFN‐γ synthesized by large numbers of CD8^+^ T cells can aggravate tissue injury.^50^ Moreover, enhanced macrophage phagocytosis in MSC‐treated mice contributed to repair of injured tissue, which is consistent with a prior report.^48^

The gastrointestinal mucosal immune system is important for maintaining host immune homeostasis.^51^ IELs regulate epithelial cell function; most express the CD8αα homodimer, and 40 to 60% are TCRγδ^+^ T cells.^52^ CD8^+^ T cells of IELs are similar to the effector memory CD8^+^ T cells that maintain the intestinal mucosal barrier.^53^ Inhibition of CD8^+^ T cells has been shown to regulate immune responses and ameliorate disease in pulmonary inflammation.^54^ We analysed the percentage of CD8^+^ T and CD8ααTCRγδ^+^ T in IELs and PPs. CD8^+^ T‐cell infiltration of the intestinal mucosa was decreased by MSCs, ameliorating injury, which is consistent with the changes in the lung. Therefore, MSCs have an immunosuppressive effect in the lung and gut in ALI.

MSCs may also promote intestinal healing by altering the intestinal bacterial community composition. Their interactions with the microbiota influence the functions of MSCs, including immunomodulation.^55^ In this study, the changes in the bacterial community were reversed by MSCs. For example, MSCs increased the Firmicutes and Bacteroidetes abundance. Lachnospiraceae, in the phylum Firmicutes, attenuated colitis in mice.^56^ MSCs improved intestinal healing by increasing the gut microbial community diversity.

The complex relationship between intestinal bacterial communities and peripheral T‐cell immunity during infection is of interest.^57^, ^58^ However, further studies are needed to determine how the intestinal microflora or activated immune response affects ALI in the complex intestinal microecological environment. We found that when antibiotics depleted the bacterial communities in ALI mice, the imbalance in intestinal microbiota aggravated lung and intestinal damage and immune response. Importantly, the damage to the lung and intestine structure was reduced by the MSC treatment. In addition, compared with the LPS group, our study demonstrated that the intestinal microbes contributed to the local host defence against pathogenic stimulus such as LPS. Immunomodulation is an important aspect of MSC therapy, so we analysed the T‐cell response of IELs over time. Interestingly, the results in antibiotic‐treated mice were similar to those in mice that were not treated with antibiotics. MSCs reduced the percentage of CD8^+^ cytotoxic T cells to alleviate inflammation in mice, which is consistent with a previous study.^59^ Indeed, the percentages of CD8^+^ T and CD8TααCRγδ^+^ T cells in our ALI mice treated with MSCs were dramatically decreased. The anti‐inflammatory activity of MSCs likely promotes recovery of ALI by modulating the synthesis of inflammatory cytokines by T cells and macrophages.^60^, ^61^ The dysregulation of the intestinal microflora increased the immune response to LPS, which may aggravate immune damage to the lung, while MSCs have the potential to serve as a therapeutic approach for lung injury due to their immunosuppressive activity.

In summary, LPS caused increased mortality, damage to the lung structure and intestinal barrier, and dysregulation of the intestinal microflora, while MSCs significantly attenuated lung and gut injury in the LPS‐induced ALI mice. Moreover, the effects of MSCs were multifactorial, involving improvement of intestinal epithelial integrity, suppression of lung and intestinal inflammation, beneficial changes in the bacterial community and enhanced lung and intestinal immunity. Our findings may have clinical implications. The widespread use of broad‐spectrum antibiotics to control lung infections may lead to an imbalance in the intestinal microbiota and a subsequent aggravation of ALI in the clinic, while early administration of MSCs may reduce the deterioration of ALI by adjusting the gut barrier and immune response. Our results provide a theoretical foundation for the protective effect of MSCs on ALI in the clinic.

## CONFLICT OF INTEREST

The authors declare no competing interests.

## AUTHOR CONTRIBUTIONS

YX and HC performed the experiments. YX, JZ, BF, XL and FL analysed the data. JZ, JL, XS and QP designed the project. YX, JY, HC and YZ analysed the data and wrote the manuscript. HC and LL designed the project and revised the manuscript.

## Supporting information

Data S1

## Data Availability

All supporting data are included in the article and its additional files.
