# Supplementary material for: Immunosuppressive effect of mesenchymal stem cells on lung and gut CD8+ T cells in lipopolysaccharide‐induced acute lung injury in mice
Source: Cell Prolif. 2021 Mar 19;54(5):e13028. doi: 10.1111/cpr.13028 (PMC8088466; doi:10.1111/cpr.13028)
Supplement: Supplementary file 1 — Data S1 [file CPR-54-e13028-s001.docx]

**Supplementary information**

**Immunosuppressive effect of mesenchymal stem cells on lung and gut CD8^+^ T cells in lipopolysaccharide-induced acute lung injury in mice**

Yanping Xu^1,2^, Jiaqi Zhu^1,2^, Bing Feng^1,2^, Feiyan Lin^1,2^, Jiahang Zhou^1,2^, Jingqi Liu^1,2^, Xiaowei Shi^1^, Xuan Lu^1,2^, Qiaoling Pan^1,2^, Jiong Yu^1,2^, Ying Zhang^1,2^, Lanjuan Li^1,2^, Hongcui Cao*^1,2,3^

1 State Key Laboratory for the Diagnosis and Treatment of Infectious Diseases, Collaborative Innovation Center for Diagnosis and Treatment of Infectious Diseases, The First Affiliated Hospital, Zhejiang University School of Medicine, 79 Qingchun Rd., Hangzhou City 310003, China

2 National Clinical Research Center for Infectious Diseases, 79 Qingchun Rd., Hangzhou City 310003, China

3 Zhejiang Provincial Key Laboratory for Diagnosis and Treatment of Aging and Physic-chemical Injury Diseases, 79 Qingchun Rd, Hangzhou City 310003, China.

***Corresponding author:**

Hongcui Cao

State Key Laboratory for the Diagnosis and Treatment of Infectious Diseases, The First Affiliated Hospital, Zhejiang University School of Medicine, 79 Qingchun Rd., Hangzhou City 310003, China. Tel: 86-571-87236451; Fax: 86-571-87236459

E-mail: hccao@zju.edu.cn

**Supplemental Materials and Methods**

**Isolation and culture of mouse MSCs**

MSCs were isolated and cultured as described by Zhu et al.^1^ Briefly, derived bone chips from 2- to 3-week-old C57BL/6 male mice were suspended in 7.5 ml C57BL/6-MSC Special Complete MEM (OriCell™ C57BL/6 MSC Complete Medium, Cyagen Biosciences, Guangzhou, China) and incubated at 37°C in a 5% CO_2_ incubator (HERAcell®150, Thermo Fisher Scientific Inc., MA). MSCs at passages 3 to 5 were considered purified and used in subsequent experiments.

**Characterization of mouse MSCs**

To induce osteogenic differentiation, MSCs were cultured in osteoinductive medium (OriCell™ C57BL6 MSC Osteogenic Differentiation Medium, Cyagen Biosciences) for 4 weeks with the appropriate supplements, followed by Alizarin Red S (Cyagen Biosciences) staining for osteocytes.

To induce adipogenic differentiation, MSCs were maintained in adipogenic culture medium (OriCell™ C57BL6 MSC Adipogenic Differentiation Medium, Cyagen Biosciences). After 4 weeks of adipogenic induction, the cultures were stained with Oil red O (Cyagen Biosciences).

The cells were stained with fluorescein PerCP-conjugated anti-mouse CD29, CD44, CD105 or PECy7-conjugated anti-mouse CD11b, CD34, CD45, CD31, and Ia antibodies. The antibodies were purchased from BioLegend (San Diego, CA). Stained MSCs were analyzed by flow cytometry (BeamCyte-1026, Beamdiag, Changzhou, China).

**Transmission electron microscopy**

Ileum samples were immediately fixed in 2.5% glutaraldehyde (Sangon Biotech), placed at 4°C for 2-4 h and embedded as described.^2^ The ultrastructure of the intestinal mucosa was analyzed using a Philips Tecnai 10 electron microscope (Philips, Eindhoven, The Netherlands).

**Immunohistochemical staining**

Ileum sections were dewaxed, rehydrated, treated with 3% H_2_O_2_, and blocked with PBS containing 5% bovine serum albumin (BSA; Sangon Biotech) at room temperature for 30 min. The sections were incubated overnight at 4°C with a primary rabbit anti-ZO-1 antibody (1:400; Invitrogen Life Technologies, Carlsbad, CA). Subsequently, the sections were incubated with a goat anti-rabbit IgG (1:50; Invitrogen Life Technologies) for 1 h at 37°C. The sections were examined using a NanoZoomer 2.0-RS scanner with the associated software.

**Real-time quantitative polymerase chain reaction**

Total RNA was harvested from intestinal segments using TRIzol Reagent (Life Technologies Corporation, Carlsbad, CA). Complementary DNA was synthesized from 1 µg total RNA using the QuantiTect Reverse Transcription Kit (Qiagen, Hilden, Germany) according to the manufacturer’s protocol. Real-time quantitative PCR (qPCR) was performed using the ABI 7500 Real-Time PCR System and the SYBR Premix Ex Taq™ II Kit (Takara Bio Inc., Shiga, Japan). The primers, synthesized by Sangon Biotech, were mouse tight junction protein 1 (ZO-1), 5’-ACTCCCACTTCCCCAAAAAC-3’ (sense primer) and 5’-CCACAGCTGAAGGACTCACA-3’ (antisense) and β-actin, 5’-ACAGGCATTGTGATGGACTC-3’ (sense)

5’-ATTTCCCTCTCAGCTGTGGT-3’ (antisense).

**FIGURE S1** Phenotype and multipotential differentiation of MSCs. (A) Flow cytometry results showed that these cells were positive for mesenchymal markers CD29, CD44 and CD105, but negative for CD11b, CD34, CD45, CD31 and MHC-la. (B) C57BL/6 mouse mesenchymal stem cells (MSCs) showed classic spindle-shaped morphology (4×). (C) Osteoblastogenesis was assayed with Alizarin Red S (× 10). (D) Adipogenesis of MSCs was stained with Oil Red O (20×). Scale bar = 100 μm.


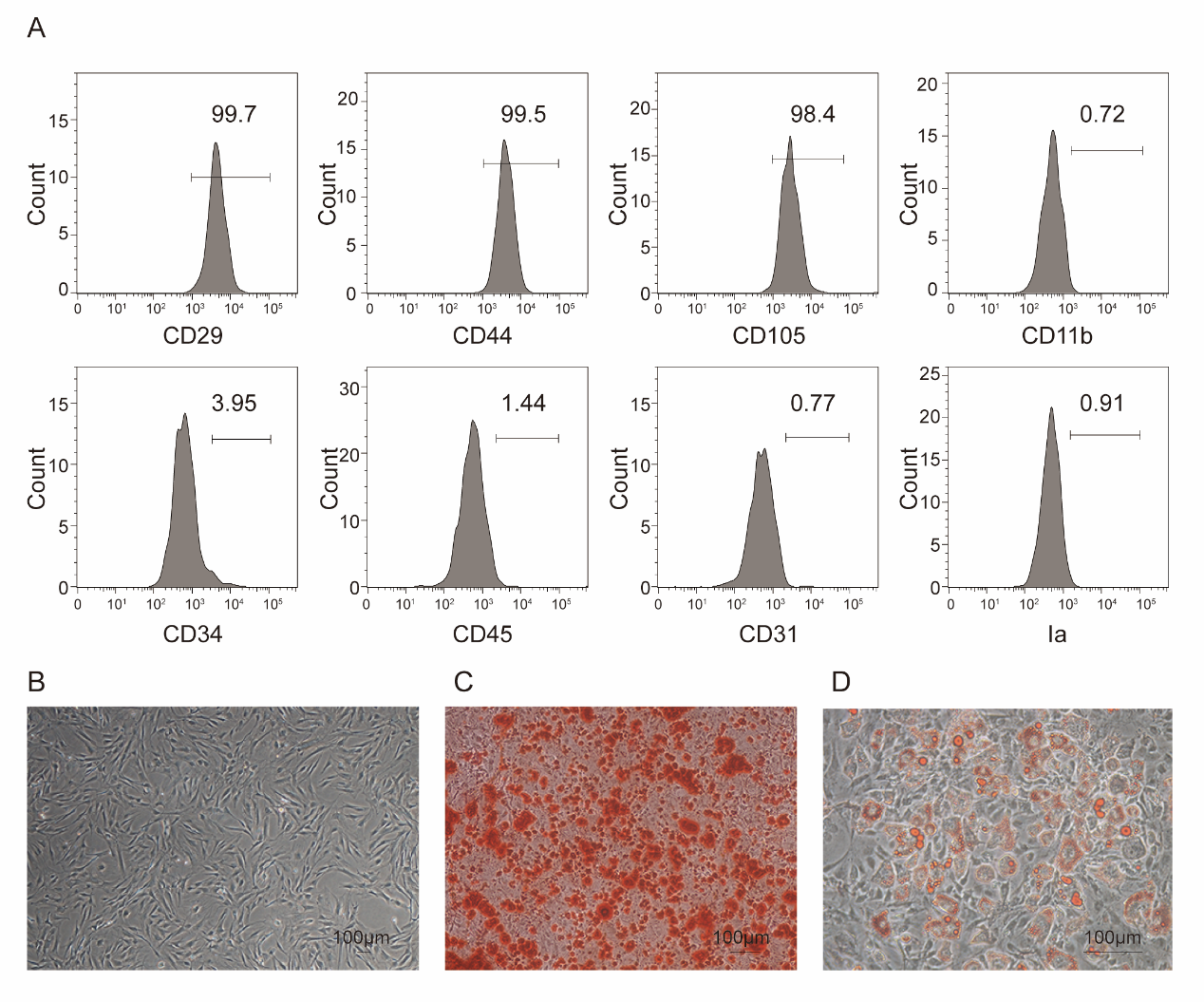


**FIGURE S2** Flow cytometric analyses of TCRγδ^+^ T cells among intestinal intraepithelial lymphocytes (IELs) and Peyer’s patches (PPs) from the indicated mice in PBS, LPS/PBS, and LPS/MSC groups. TCRγδ^+^ T cells were mostly expressed in intestinal epithelial cells and expressed at a low level in PPs in PBS group.

**
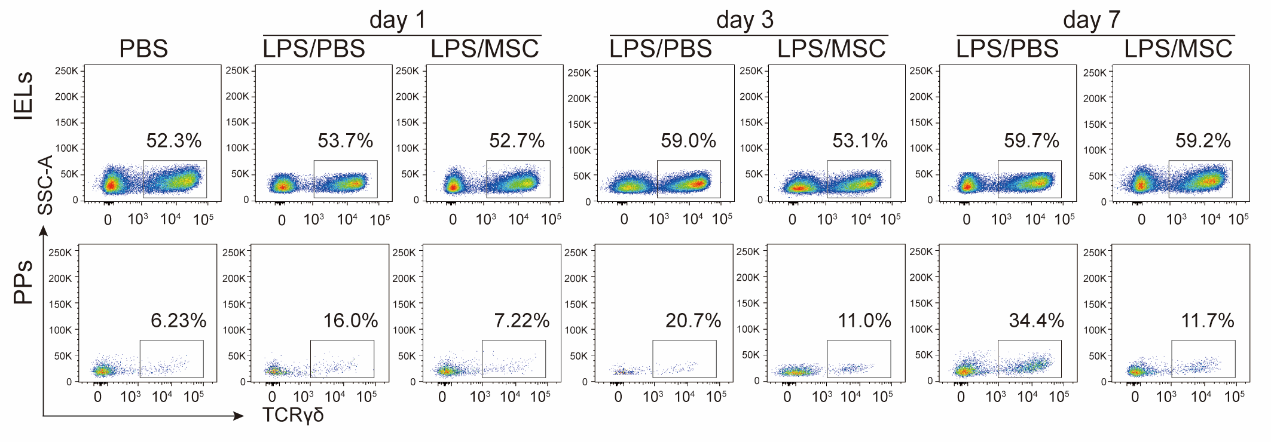
**

**FIGURE S3** Rarefaction curves for the observed species in bacterial communities from the PBS, LPS/PBS, and LPS/MSC groups. N = 8 per group. L: lipopolysaccharide (LPS)-induced acute lung injury (ALI) with PBS; M: LPS-induced ALI mice treated with MSCs. 1 d, 3 d and 7 d indicate 1 day, 3 days and 7 days after LPS treatment, respectively.


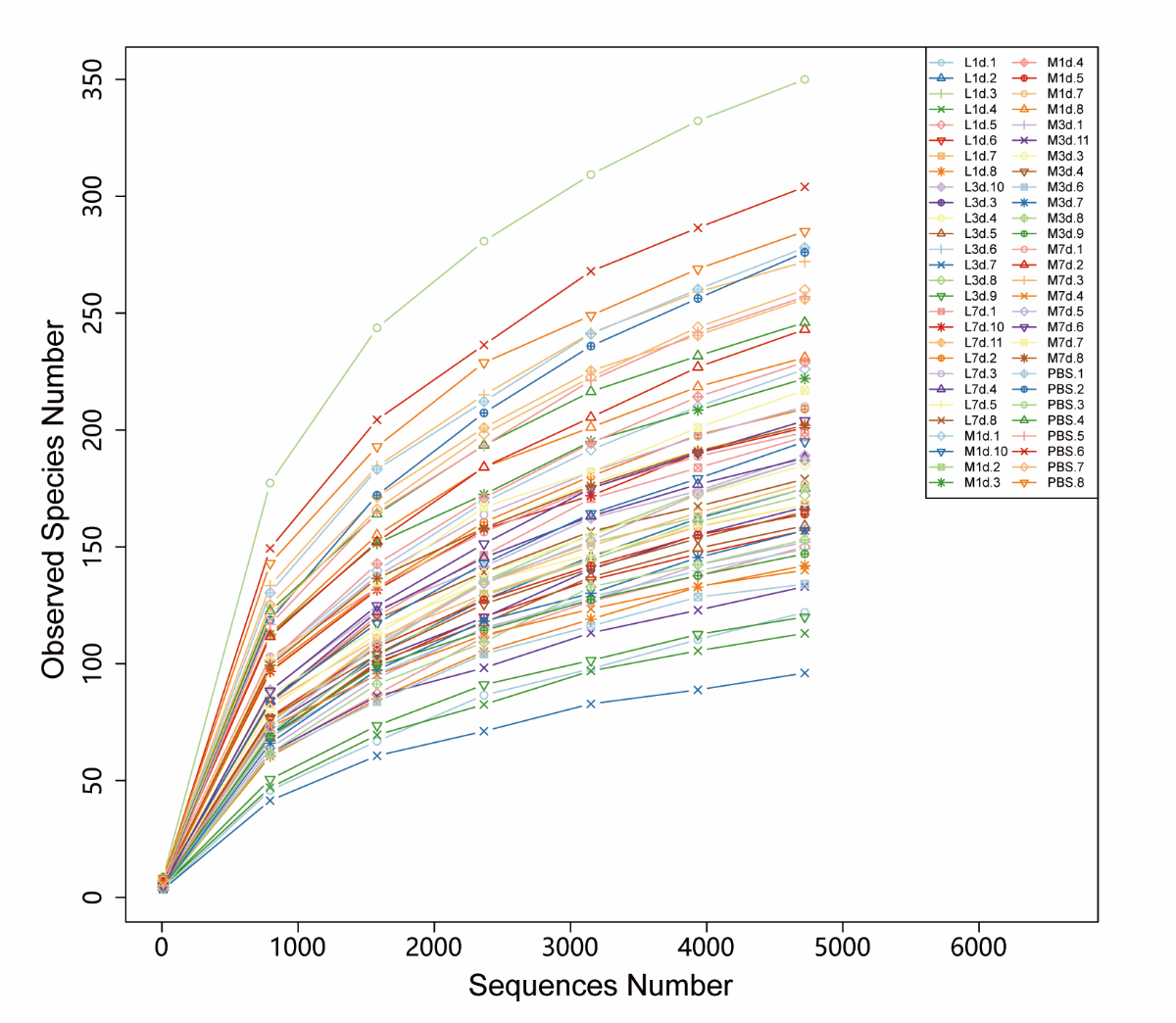


**FIGURE S4** Diversity and composition of the intestinal bacterial community in mice with acute lung injury. (A) Analysis of similarity (ANOSIM) revealed a significant difference in beta-diversity (p < 0.01) from PBS, LPS/PBS, LPS/MSC groups. (mean ± SEM, n = 8). (B) Numbers of observed species of 16 S rRNA gene sequences at days 1, 3, and 7 in PBS, LPS/PBS, LPS/MSC groups. (mean ± SEM, n = 8). ***p < 0.001 by Student’s *t*-test. (C) Venn diagram generated according to the operational taxonomic unit (OTU) clustering of the three group at day 1, each number represents the number of bacterial species, either shared or unique to groups. (D) Linear discriminant analysis (LDA) effect size (LEfSe) was based on 16 S rRNA gene sequences at days 1, 3, and 7 (α = 0.01, LDA score > 4.0). Histogram of the LDA scores computed for differentially abundant bacterial taxa in LPS/PBS, LPS/MSC groups (n = 8). L: lipopolysaccharide (LPS)-induced acute lung injury (ALI) with PBS; M: LPS-induced ALI mice treated with MSCs. 1 d, 3 d and 7 d indicate 1 day, 3 days and 7 days after LPS treatment, respectively.


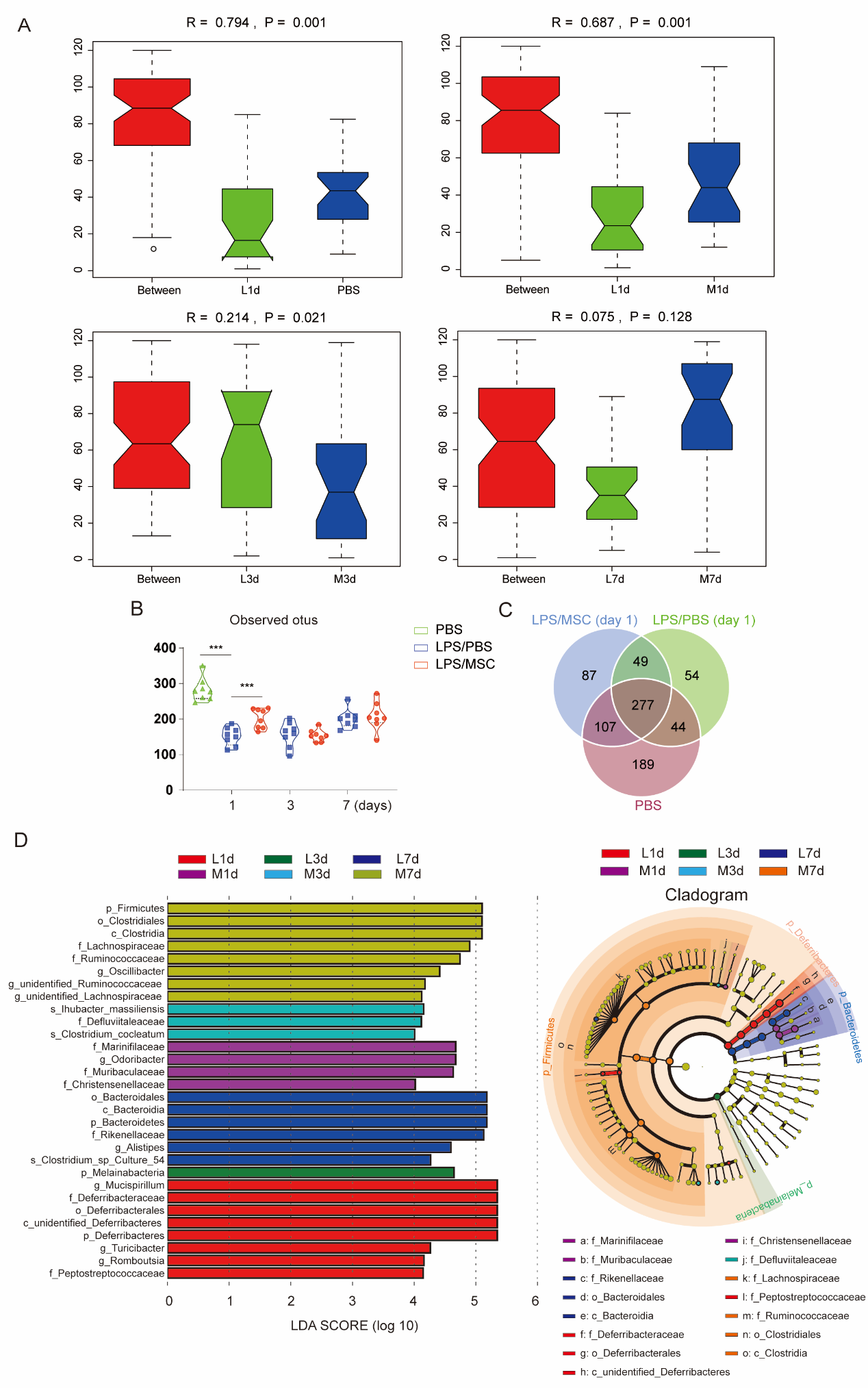


**TABLE S1 Numbers of raw reads, clean reads, and bases; average read lengths; good coverage estimations; and operational taxonomic units as determined via PacBio sequencing analyses.**

| Sample | Raw reads | Clean reads | Base (nt) | AvgLen (nt) | Goods coverage | Observed otus |
| --- | --- | --- | --- | --- | --- | --- |
| M1d.1 | 16967 | 16635 | 24328156 | 1462 | 0.981 | 226 |
| M1d.2 | 16783 | 16616 | 24379752 | 1467 | 0.988 | 175 |
| M1d.3 | 16203 | 15393 | 22573224 | 1466 | 0.985 | 222 |
| M1d.4 | 21126 | 20917 | 30669807 | 1466 | 0.98 | 229 |
| M1d.5 | 20528 | 14528 | 21243841 | 1462 | 0.99 | 164 |
| M1d.7 | 19550 | 17987 | 26469098 | 1471 | 0.987 | 177 |
| M1d.8 | 18149 | 17790 | 26037595 | 1463 | 0.985 | 231 |
| M1d.10 | 23536 | 17042 | 24965638 | 1464 | 0.984 | 195 |
| M3d.1 | 22483 | 17177 | 25192631 | 1466 | 0.988 | 152 |
| M3d.3 | 21131 | 19478 | 28577190 | 1467 | 0.984 | 185 |
| M3d.4 | 15666 | 15353 | 22549427 | 1468 | 0.988 | 165 |
| M3d.6 | 10961 | 9022 | 13207806 | 1463 | 0.991 | 134 |
| M3d.7 | 21038 | 20830 | 30580492 | 1468 | 0.986 | 157 |
| M3d.8 | 17178 | 17019 | 24998209 | 1468 | 0.988 | 153 |
| M3d.9 | 18662 | 18103 | 26555038 | 1466 | 0.991 | 147 |
| M3d.11 | 18786 | 14984 | 21989132 | 1467 | 0.989 | 133 |
| M7d.1 | 18850 | 12982 | 19036046 | 1466 | 0.988 | 199 |
| M7d.2 | 17587 | 16709 | 24515182 | 1467 | 0.98 | 243 |
| M7d.3 | 22170 | 14841 | 21722845 | 1463 | 0.984 | 272 |
| M7d.4 | 18525 | 17785 | 26156756 | 1470 | 0.992 | 140 |
| M7d.5 | 10569 | 6559 | 9584375 | 1461 | 0.981 | 187 |
| M7d.6 | 19086 | 18887 | 27620741 | 1462 | 0.984 | 204 |
| M7d.7 | 24671 | 16507 | 24156756 | 1463 | 0.983 | 217 |
| M7d.8 | 14046 | 9360 | 13702738 | 1463 | 0.986 | 202 |
| PBS.1 | 21953 | 16617 | 24383270 | 1467 | 0.98 | 278 |
| PBS.2 | 22538 | 16069 | 23570728 | 1466 | 0.978 | 276 |
| PBS.3 | 18838 | 13154 | 19307257 | 1467 | 0.98 | 350 |
| PBS.4 | 21719 | 21294 | 31249778 | 1467 | 0.985 | 246 |
| PBS.5 | 24809 | 17222 | 25355283 | 1472 | 0.98 | 257 |
| PBS.6 | 16714 | 16405 | 24076056 | 1467 | 0.98 | 304 |
| PBS.7 | 16377 | 16053 | 23572758 | 1468 | 0.979 | 260 |
| PBS.8 | 15622 | 15322 | 22464654 | 1466 | 0.982 | 285 |
| L1d.1 | 19950 | 19352 | 28585010 | 1477 | 0.987 | 122 |
| L1d.2 | 18322 | 14130 | 20783651 | 1470 | 0.985 | 175 |
| L1d.3 | 19240 | 17702 | 26058257 | 1472 | 0.984 | 187 |
| L1d.4 | 15311 | 12111 | 17800870 | 1469 | 0.991 | 113 |
| L1d.5 | 19373 | 18986 | 27927735 | 1470 | 0.988 | 150 |
| L1d.6 | 16061 | 13144 | 19254186 | 1464 | 0.989 | 157 |
| L1d.7 | 12488 | 9681 | 14135034 | 1460 | 0.988 | 168 |
| L1d.8 | 15234 | 13158 | 19370207 | 1472 | 0.989 | 142 |
| L3d.3 | 22492 | 22056 | 32424733 | 1470 | 0.985 | 167 |
| L3d.4 | 19722 | 19293 | 28419268 | 1473 | 0.986 | 202 |
| L3d.5 | 10005 | 7804 | 11401601 | 1460 | 0.988 | 159 |
| L3d.6 | 14213 | 11395 | 16737469 | 1468 | 0.99 | 149 |
| L3d.7 | 14405 | 12550 | 18438744 | 1469 | 0.992 | 96 |
| L3d.8 | 17401 | 17060 | 25000697 | 1465 | 0.986 | 172 |
| L3d.9 | 19012 | 18660 | 27264036 | 1461 | 0.99 | 120 |
| L3d.10 | 17503 | 16278 | 23847274 | 1465 | 0.985 | 189 |
| L7d.1 | 24492 | 17065 | 24913733 | 1459 | 0.985 | 197 |
| L7d.2 | 15815 | 15499 | 22681707 | 1463 | 0.985 | 209 |
| L7d.3 | 19558 | 13014 | 19015726 | 1461 | 0.986 | 210 |
| L7d.4 | 20242 | 14227 | 20797457 | 1461 | 0.988 | 188 |
| L7d.5 | 22022 | 17104 | 25040897 | 1464 | 0.99 | 168 |
| L7d.8 | 20643 | 20243 | 29701418 | 1467 | 0.987 | 179 |
| L7d.10 | 17930 | 11244 | 16455373 | 1463 | 0.985 | 201 |
| L7d.11 | 25096 | 15412 | 22565859 | 1464 | 0.982 | 256 |

L: lipopolysaccharide (LPS)-induced acute lung injury (ALI) with PBS; M: LPS-induced ALI mice treated with MSCs. 1 d, 3 d and 7 d indicate 1 day, 3 days and 7 days after LPS treatment, respectively.

**REFERENCES**

1. Zhu H, Guo ZK, Jiang XX, et al. A protocol for isolation and culture of mesenchymal stem cells from mouse compact bone. *Nature protocols*. 2010;5(3):550-560.

2. Dong XT, Feng XD, Liu JQ, et al. Characteristics of intestinal microecology during mesenchymal stem cell-based therapy for mouse acute liver injury. *Stem Cells International*. 2019.
